# Detection of Norovirus from Berries in Serbia by Digital PCR and NGS

**DOI:** 10.3390/foods14183257

**Published:** 2025-09-19

**Authors:** Branko Velebit, Vesna Janković, Marina Velebit, Tamara Bošković, Milica Jovanović, Dapeng Wang, Dunja Mišić

**Affiliations:** 1 Institute of Meat Hygiene and Technology, 11040 Belgrade, Serbia; vesna.jankovic@inmes.rs (V.J.); dunja.misic@inmes.rs (D.M.); 2 Agrosava, 11070 Belgrade, Serbia; mvelebit@agrosava.com; 3 Ministry of Agriculture, Forestry and Water Management, 11070 Belgrade, Serbia; tamara.boskovic@minpolj.gov.rs; 4 Department of Microbiology, University Clinical Center of Serbia, 11000 Belgrade, Serbia; mijovan@eunet.rs; 5 School of Agriculture and Biology, Shanghai Jiao Tong University, Shanghai 200240, China; dapengwang@sjtu.edu.cn

**Keywords:** norovirus, berry, fruit, food safety, digital PCR, next generation sequencing

## Abstract

Human norovirus (HuNoV), a primary cause of foodborne illness, is frequently transmitted through contaminated berries. Serbia is a global producer of raspberries and other berries, yet data on HuNoV prevalence and genogroup diversity are limited. This study aimed to assess the prevalence, viral load, and genotypes of HuNoV circulating in raspberries and blackberries marketed in Serbia. A total of 450 berry samples were collected in 2023 from orchards, cold storage facilities, local markets, and roadside vending stands. Norovirus RNA was extracted using a modified ISO 15216-2 protocol. RT-qPCR positive samples were subsequently quantified by digital RT-PCR (RT-dPCR). Genotyping employed next-generation sequencing (NGS) of genome encoding major and minor capsid proteins, supplemented by Sanger sequencing. Nineteen samples (4.2%) tested positive for HuNoV, including four GI and fifteen GII strains, with higher prevalence in frozen (11.1%) than fresh berries (2.0%). Viral loads ranged from 34–105 gc/g for GI and 23–658 gc/g for GII, with medians of 72 and 153 gc/g, respectively. Genotypes GI.6, GII.2, GII.4, and GII.7 were identified, each represented by more than two sublineages and multiple introduction events detected by phylogenetic analysis. RT-dPCR provided precise quantification, while NGS enabled genotype characterization, together supporting enhanced surveillance, risk assessment, and management of norovirus contamination in berries destined for domestic and international markets.

## 1. Introduction

Epidemiological investigations and global surveillance data have increasingly pointed to berries [1,2,3,4,5,6,7,8] and leafy greens [9,10,11,12,13,14,15,16] as important vehicles in the transmission of human norovirus (HuNoV). The Food and Agriculture Organization (FAO) ranks fecally contaminated berries as the second highest microbiological threat to consumer health [16]. From 1983 to 2018, HuNoV and hepatitis A virus (HAV) together were implicated in over 72% of berry-associated outbreaks and more than 80% of illnesses linked to these fruits worldwide, contributing to an estimated 125 million cases of foodborne disease and 35,000 deaths [5,17]. The public health impact of such outbreaks has been magnified in high-income countries, where urbanization and greater purchasing power have promoted dietary shifts toward minimally processed, nutrient-dense “superfruits.” At the same time, modern transport systems allow rapid shipment of berries from regions with inadequate hygiene practices, creating conditions for large-scale, cross-border outbreaks. Serbia illustrates this risk: it produces nearly one quarter of the world’s raspberries, with almost all intended for export [18,19]. Despite hygiene measures, data from the European Rapid Alert System for Food and Feed (RASFF) show that between 2006 and 2024, contaminated Serbian berries were associated with 33 of 132 reported norovirus contamination events. Most involved were frozen raspberries (75.7%), although blackberries, strawberries, and mixed forest fruits were also implicated [20]. Both GI and GII HuNoV genogroups were detected in these events, highlighting the vulnerabilities inherent in international trade of frozen berries.

Transmission of norovirus occurs through the fecal–oral route, either person to person or via contaminated food and water. Infected individuals can shed extremely high quantities of norovirus, with concentrations reaching up to 10^10^ particles per gram of stool, whereas the infectious dose for disaggregated HuNoV may be as low as 18 particles [21,22,23,24]. As a result, even a relatively small portion of berries (≈100 g) contaminated at levels below 100 genome copies per gram (gc/g) may be sufficient to cause illness. Although HuNoV does not replicate in food, it retains infectivity under a wide range of temperatures and pH conditions but resists many conventional chemical disinfectants [16,24]. HuNoV produces no perceptible deterioration of the berries, and viral contamination has no effect on the food’s organoleptic properties rendering these microorganisms particularly challenging to control [21,25].

Reliable detection of HuNoV in berries remains a major challenge. The number of viral particles on fruit surfaces is typically low, while inhibitory compounds such as polyphenols and anthocyanins interfere with molecular assays. Analysis is further aggravated by typically quite low recovery rates which range from 1% to 10%. This significantly heightens the probability of false negative results when detecting trace amounts of virus in food commodities that approach the threshold of the human infectious dose [7,26,27]. Currently, the reverse transcription–quantitative PCR (RT-qPCR) is the gold standard approach for detection of enteric viruses, but its accuracy is limited by matrix effects and sensitivity to inhibitors [28] and variation in specificity and sensitivity [29]. Moreover, RT-qPCR assays for norovirus on clinical and environmental samples have a significantly reduced genome amplification efficiency [30]. Next, in RT-qPCR Cq value is translated to correspond to RNA copies per g or sample of berries, which is further used as proxy for numbers of viruses [31]. Finally, RT-qPCR is unable to differentiate between infectious from non-infectious viral particles [32]. This limitation is particularly challenging since monitoring systems rely primarily on RT-qPCR, which may inflate perceived risk if viral RNA remains detectable after loss of infectivity.

Digital RT-PCR (RT-dPCR), a third-generation PCR method, could enhance viral RNA detection and quantification by partitioning reactions into ≈26,000 nanoliter compartments. Each undergoes parallel amplification and is classified as positive or negative, allowing absolute quantification via Poisson statistics [33,34]. In parallel, next-generation sequencing (NGS) and Nanopore and PacBio sequencing have emerged as powerful tools in the whole-genome sequencing (WGS) approach to characterize viral diversity and trace outbreak transmission routes. Recent work has shown that amplification of long capsid gene fragments, coupled with NGS, can provide sufficient coverage to detect HuNoV in frozen raspberries, illustrating the promise of this method for food safety investigations [7].

This study addresses the existing knowledge gap regarding the HuNoV genotype diversity and contamination levels in Serbian berries in an effort to strengthen future global outbreak investigations, contribute to the establishment of evidence-based regulatory limits, and enhance the accuracy of quantitative microbial risk assessment models.

## 2. Materials and Methods

### 2.1. Sample Collection

**Figure 1:**
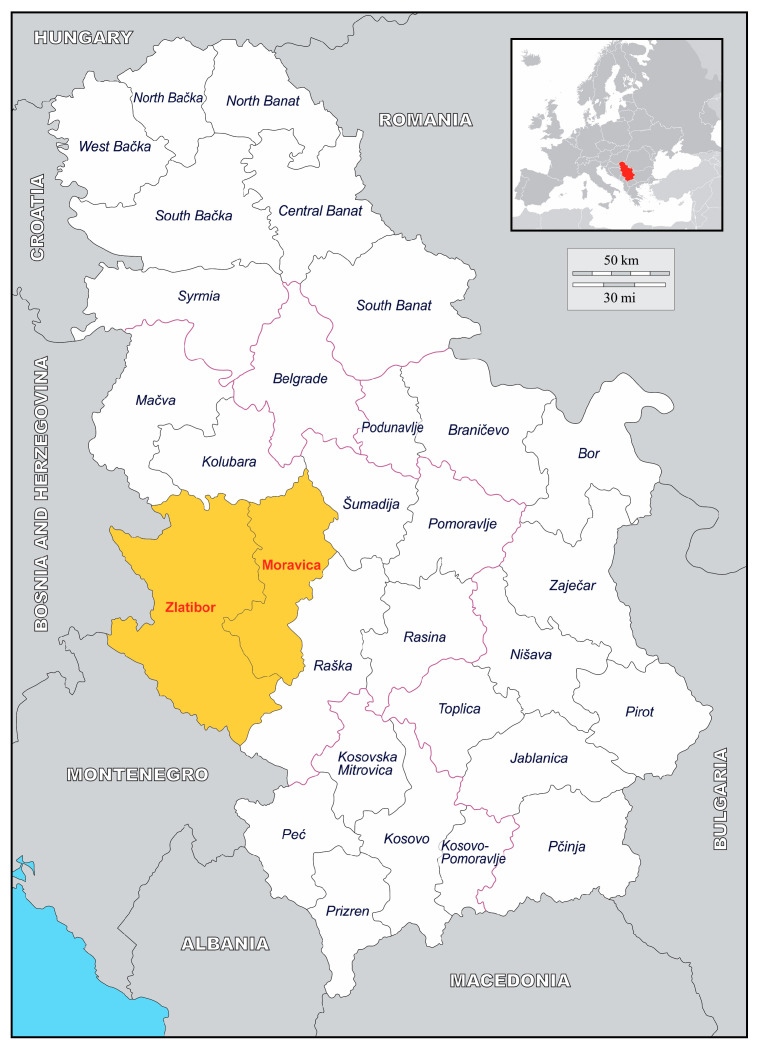
A location map highlighting the study area, shown in amber.

A total of 450 berry samples (358 raspberries and 92 blackberries) were collected in 2023 in Zlatibor and Moravica districts (Western Serbia), where most of the berry orchard farms are situated and where most of the contaminated berries involved in RASFF events originated from (Figure 1). Samples were taken during the regular fruit harvesting season between June to August. Sampling sites included 9 orchard farms, 6 large-scale cold storage facilities, 6 berry vending stands frequented by tourists along the roadside and 4 local markets situated in community centers. The surveyed area encompassed 1176 km^2^, extending in a northwest–southeast orientation, approximately delineated by the geographical coordinates 43°58′38″ N, 19°35′17″ E and 43°33′53″ N, 20°16′13″ E.

Each sample comprised approximately 100 g of either fresh or frozen berries. The sampling scheme consisted of six samples of fresh berries collected from each growing farm, six samples of frozen berries obtained from each cold storage facility, six samples of fresh berries sourced from each local market, and six samples of fresh berries gathered from each selling point. Samples were transported to the laboratory while maintaining temperature-controlled conditions.

### 2.2. Norovirus Elution, Viral RNA Extraction and PCR Inhibitor Removal

Norovirus was eluted according to the protocol described in the ISO 15216-2:2019 [35] although some modifications were employed to increase virus recovery and to reduce presence of inhibitors. The comparison of extraction efficiencies, inhibition, and Cq values between the ISO method and the modified ISO method is presented in Supplementary Table S1. Briefly, 25 g of berries were placed in a 400 mL Stomacher bag with mesh filter compartment (Interscience, Saint Nom la Bretêche, France), and 10 μL (corresponding to 10^6^ genomic copies) of the Mengovirus strain vMC0 (ATCC VR-1597) suspension was added to the berry samples and incubated at room temperature for 10 min. Elution was performed with the addition of 120 mL of 0.1 M Tris-HCl, 0.05 M glycine, 2% polyvinylpyrrolidone, and 1% beef extract buffer (TGBE) at pH 9.2 with 500 μL of pectinase (≥3.800 units/mL; Sigma, St. Louis, MO, USA) on a shaking platform (IKA Werke, Staufen, Germany) for 30 min at 180 rpm and occasionally checking the pH value and readjusting to 9.5 with 10 M NaOH if necessary. After centrifugation (10,000× *g*, 30 min) to pellet berry debris, the supernatant was collected and the pH adjusted to 7. Virus was concentrated by overnight precipitation with PEG 8000 (Sigma) at a final concentration of 10% (*w*/*v*) with 1.5 M NaCl at 4 °C, pelleted by centrifugation at 10,000× *g* for 30 min at 4 °C, and resuspended in 500 μL of PBS and 500 μL of a chloroform/butanol mixture (1:1, *v*/*v*). After centrifugation (10,000× *g*, 30 min) supernatant was mixed with Vertrel XF (DuPont, Wilmington, NC, USA), and after final centrifugation, aqueous phase (450–500 μL) was resuspended in 2 mL of Nuclisense Lysis Buffer (Biomerieux, Marcy-l’Étoile, France). The RNA was isolated per manufacturer’s instructions and eluted with 100 μL of elution buffer.

A two-step viral RNA purification was performed. Firstly, the eluted viral RNA was purified using the OneStep PCR Inhibitor Removal Kit (Zymo Research, Irvine, CA, USA), according to the manufacturer’s instructions. In the second step, viral RNA was further processed by the RNA Clean & Concentrator-25 columns (Zymo Research, Irvine, CA, USA). Briefly, RNA sample was mixed with RNA Binding Buffer and ethanol and pipetted in IIC purification column, centrifuged for 30 s at 800× *g*. Next, RNA Prep Buffer was added and the column was once again centrifuged (same conditions). After washing and centrifugation, pure and concentrated RNA was eluted in 60 µL of RNA-se-free water and stored at −80 °C until RT-qPCR analysis.

### 2.3. RT-qPCR and Digital RT-PCR

Initial screening for noroviruses in berry samples was conducted using a one-step RT-qPCR, in accordance with ISO 15216-2:2019 [35]. The assay was performed with the TaqMan Fast Virus 1-Step Master Mix (Thermo Fisher Scientific, Waltham, MA, USA) on an AriaMx Real-Time PCR System (Agilent Technologies, Santa Clara, CA, USA). Each reaction contained 500 nM forward primer, 900 nM reverse primer, and 150 nM probe (Table 1). For each sample, a total of 25 μL reaction mix was prepared with 20 μL of reagents and 5.0 μL of the sample. RT-qPCR was performed with RT at 50 °C for 5 min, inactivation of the reverse transcriptase and activation of the DNA polymerase at 95 °C for 20 s, 45 cycles of denaturation at 95 °C for 3 s, and annealing/elongation at 60 °C for 30 s. Results were analyzed with the Agilent Aria software, version 2.1.

RT-qPCR extraction efficiency was assessed solely as a quality assurance measure and was not applied to adjust the final test results. Efficiency was determined by calculating the recovery of the process control virus, Mengovirus, relative to a Mengovirus RNA standard curve. Recovery was estimated using the formula Mengovirus recovery (%) = 10(ΔCq/m) × 100, where ΔCq represents the difference between the Cq value of the sample RNA and that of the undiluted Mengovirus RNA standard, and “m” denotes the slope of the Mengovirus RNA standard curve. To evaluate potential RT-qPCR inhibition, an external control (EC), consisting of in vitro-synthesized viral RNA transcripts, was included. These external control RNA stocks were generated as described in Annex G of the ISO 15216-2:2019 [35]. Inhibition was assessed as acceptable if the Cq value of the undiluted sample RNA + EC RNA was <2.00 greater than the Cq value of the water + EC RNA well.

Norovirus-positive RNA samples identified by RT-qPCR were subsequently quantified using digital RT-PCR. HuNoV quantification was performed on the QIAcuity One dPCR System (QIAGEN). A QIAcuity One-Step Advanced PCR Kit (Qiagen, Hilden, Germany) was used for one-step RT-dPCR. The singleplex one-step RT-dPCR reaction used a total volume of 44 μL, which consisted of 11 μL of 4× One-Step Advanced Probe Master Mix, 0.44 μL of 100× One-Step Advanced RT Mix, 500 nM of forward primer, 900 nm of reverse primer, 250 nM of probe, and 11 μL of RNA template. The RT-dPCR mix was prepared in a pre-plate and then transferred into the 24-well 26 K Nanoplate. The QIAcuity One fully automated workflow included: the priming and rolling step in order to generate and seal the single partitions; the reverse transcription step, 47 °C; the amplification step, 95 °C for 2 min, 95 °C for 15 s and 53 °C for 60 s for 45 cycles; and the final imaging step, done by reading the FAM channel of HuNoV GI and HEX channel of HuNoV GII, respectively. Data were analyzed using the QIAcuity Suite Software V3.1. The RT-dPCR assays were performed using standardized settings for threshold and baseline.

Raw RT-dPCR data were exported from the Qiacuity One platform. The concentration of target RNA in the undiluted RNA extract (in genome copies/μL) was calculated as four times the concentration measured in the RT-dPCR reaction to account for dilution in the reaction setup (i.e., 11 μL RNA in a total volume of 44 μL). Viral load per berry sample was then determined using the formula: Viral load of berry (genome copies/g) = copies/μL RNA × total RNA extracted (μL) × 1/total grams of berry sampled. RT-dPCR inhibition was calculated as a percentage using the following formula: 1 − ((genome copies (sample + EC)/genome copies (external amplification control + water)) × 100%).

As no standard or guideline currently defines the LOD for RT-dPCR, the probability of detection (POD) approach was applied to estimate the LOD95. Serial dilutions of in vitro-transcribed viral RNA (EC) in raspberry RNA extracts (1–1000 gc/reaction), previously quantified using the QIAcuity One dPCR system, were tested. A minimum of 10 replicates per dilution level were analyzed in independent RT-dPCR runs. Data were processed using PODLOD software, version 12 [41]. The limit of quantification (LOQ) was defined as the lowest concentration at which the coefficient of variation (CV) of back-calculated replicate concentrations was below 35%, calculated as CV = 100 × (SD/mean) [42].

### 2.4. Sequencing of HuNoV

Sequencing and genotyping of HuNoV RNA were performed following protocols described by Raymond et al. [7] and Parra et al. [43]. Samples that tested positive for HuNoV GI or GII by RT-qPCR were subjected to multiplex long-range two-step RT-PCR (MLR2), generating ≈2.2–2.5 kb amplicons spanning the complete ORF2 and ORF3 regions of the viral genome. The resulting amplicons were subsequently analyzed using the next-generation sequencing (NGS) technique. Briefly, complementary DNA (cDNA) was synthesized from the viral RNA by two-step RT-PCR using 5 μM of reverse Tx30SXN primer (GACTAGTTCTAGATCGCGAGCGGCCGCCCTTTTTTTTTTTTTTTTTTTTTTTTTTTTTT) and the Maxima H Minus First Strand cDNA Synthesis Kit (Thermo Fisher Scientific, Waltham, MA, USA), following manufacturer’s recommendations. Amplification of the genome fragments was performed in a AB2720 thermocycler (Applied Biosystem, Foster City, CA, USA) using 5 μL of the RT reaction, a set of primers that target the conserved regions of the 5′- and 3′-end of GI and GII noroviruses (QNIF4: CGCTGGATGCGNTTCCAT and QNIF2: ATGTTCAGRTGGATGAGRTTCTCWGA, and Tx30SXN, each at 500 nm, respectively), and the LongAmp Hot Start polymerase kit (New England Biolabs, Ipswich, MA, USA) according to the manufacturer’s instructions. After an initial denaturation step at 94 °C for 30 s, the cDNA was amplified with 35 cycles of 15 s at 94 °C, 30 s at 60 °C and 90 s at 65 °C followed by a final elongation at 65 °C for 10 min. Amplicons were subjected to electrophoresis on 1.5% agarose gel containing a SYBR Safe DNA Gel Stain (Thermo Fisher Scientific, Waltham, MA, USA) at 100 V for 45 min, visualized under UV light on the Enduro GDS Touch Gel Documentation System (Labnet, Edison, NJ, USA), excised and purified with the QIAquick Gel Extraction Kit (Qiagen, Hilden, Germany).

Purified MLR2 amplicons were quantified using the Qubit dsDNA HS Assay kit (Thermo Fisher Scientific, Waltham, MA, USA) and concentrations were adjusted to 200 pg/μL DNA. For each sample a 300 bp fragment library (already ligated with the bar-coded adapter) was constructed using the NEBNext Ultra II RNA Library Prep Kit for Illumina (New England Biolabs, Ipswich, MA, USA) according to the manufacturer’s instructions. Obtained cDNA libraries were purified using Agencourt AMPure XP magnetic beads (Beckman Coulter, Brea, CA, USA) and their fragment sizes were confirmed using the Agilent Bioanalyzer 2100 and High Sensitivity DNA assay (Agilent Technologies, Santa Clara, CA, USA). Paired-end Illumina sequencing was performed on a MiSeq sequencer employing 2 × 250 bp paired-end chemistry (MiSeq Reagent Kit v3) according to manufacturer instructions (Illumina, San Diego, CA, USA).

Raw sequencing data were processed using MiSeq Reporter v2.6 to generate demultiplexed FASTQ files. Reads were trimmed for adapters and bases with a Phred quality score < 30 using Trimmomatic v0.39, and sequences < 50 bp after trimming were discarded. Filtered viral reads were aligned against a curated norovirus reference panel comprising N = 4321 complete genomes deposited in GenBank as of 11 July 2025. Both reference-guided mapping and de novo assembly were performed. Contigs shorter than 250 nt were excluded from downstream analysis. Resulting contigs were compared against GenBank using BLASTn (v. 2.17.0) with a minimum match size of 100 nt and an E-value threshold of 1 × 10^−8^. Determination of Norovirus genotypes was based on capsid sequence and Norovirus Typing Tool (RIVM, Bilthoven, The Netherlands) was used for genotyping [44]. Resulting sequences were submitted to the GenBank database, with the accession numbers OR794161-PQ844641.

When NGS sequencing of long MLR2 amplicons was unsuccessful, conventional Sanger sequencing of partial ORF1/ORF2 amplicons was attempted using the One-Step RT-PCR Kit (Qiagen, Hilden, Germany) with primers GISKF/GISKR and GIISKF/GIISKR [45]. The resulting amplicons were sequenced at Microsynth, Switzerland.

### 2.5. Phylogenetic Analysis

Sequence editing and multiple alignments were performed using CLC Genomic Workbench v14 (Qiagen, Hilden, Germany). The HuNoV viral genome sequences obtained in this study and the related reference full genome norovirus sequences retrieved from GenBank database were aligned using ClustalW. Maximum likelihood-based phylogenetic trees were constructed using initial trees for the heuristic search obtained by applying the Neighbor-Joining (NJ) method. The evolutionary history was inferred by using the maximum likelihood method employing the Tamura-Nei 4-parameter model. Bootstrap analysis using a total of 1000 replicates was employed to verify reliability of the trees. Evolutionary analyses were conducted in MEGA12 [46].

### 2.6. Statistical Analysis

The prevalence of norovirus in berries and their 95% confidence intervals were calculated using the Pearson–Klopper method. The Chi-square test with Yates’ correction was executed to compare the significance (*p*-value) of differences between positive and negative results. All statistical calculations were performed using the GraphPad Prism 10 (GraphPad Software, Boston, MA, USA).

## 3. Results

### 3.1. Detection and Quantification of HuNoV RNA

Out of the total berries analyzed by RT-qPCR (n = 450), 108 samples (24%) corresponded to frozen fruits and 342 (76%) to fresh fruits. Out of 450 samples, nineteen (4.2%, 95% CI: 2.6–6.5%) tested positive for viral detection by RT-qPCR screening (four for HuNoV GI and fifteen for HuNoV GII), of which there were three blackberry and sixteen raspberry samples. Among the frozen fruits, 11.1% (12/108) tested positive (95% CI: 5.9–18.6%), detecting HuNoV GI in three (2.8%) samples and HuNoV GII in nine samples (8.3%). For fresh fruit, 2.0% (7/342) tested positive (95% CI: 0.8–4.2%), detecting HuNoV GI in one (0.3%) sample and HuNoV GII in six samples (1.7%). Significant differences were found in prevalence between fresh and frozen berries (*p* < 0.001). The majority of HuNoV RT-qPCR-positive samples (63.2%) were detected in frozen berries sourced from cold storage facilities. A smaller proportion (31.6%) of positive samples were found in fresh berries collected from orchard farms, while a single positive sample (5.3%) was identified in fresh raspberries obtained from a local market situated in a community center. No positive samples were detected in berries collected from retail points along tourist routes in either Zlatibor or Moravica counties.

**Figure 2:**
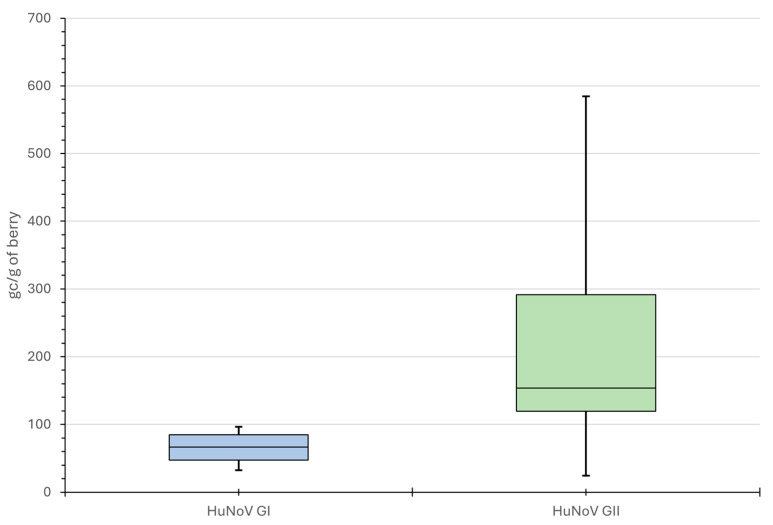
Box plot showing HuNoV GI and GII viral load (gc/g of berry) by RT-dPCR. The horizontal line represents the median value, the box is the interquartile range and the lower and upper whiskers represent the 5% and 95% percentile ranges.

No berry samples were shown to be inhibitory, as determined by both the RT-qPCR (<2 Cq) and RT-dPCR assays (<75%) and HuNoV was detected mainly in undiluted RNA extracts. Virus extraction efficiencies, as determined by the recovery of Mengovirus, ranged between 1.3% and 11.6%. Using RT-dPCR, HuNoV viral load in berries ranged from 34 to 105 gc/g for HuNoV GI (n = 4) and 23 to 658 gc/g of berry for HuNoV GII (n = 15) (Table 2). The median HuNoV GI and GII concentrations were 72 gc/g and 153 gc/g of berry, respectively (Figure 2). Some samples that screened positive for GII Cq > 38 using RT-qPCR were negative using RT-dPCR.

Limit of Blank (LOB) for HuNoV GI and GII RT-dPCR assays was calculated using a non-parametric approach analyzing N = 30 blank samples to reach a confidence level of 95% [47]. LOB for HuNoV GI and GII was 0.046 gc/µL and 0.00 gc/µL, respectively. Back-calculated RT-dPCR LOD95 for HuNoV GI and HuNoV GII was 8.8 gc/g and 3.1 gc/g of berry, respectively, while LOQ for HuNoV GI and HuNoV GII was 13 gc/µL and 18 gc/µL. There was a significant (*p* < 0.001) statistical difference between the RT-qPCR and RT-dPCR LOD95 for HuNoV GI and HuNoV GII.

### 3.2. Genotyping and Phylogenetic Analysis

The nineteen RNA extracts that were positive for HuNoV GI and GII by RT-qPCR were subjected to MLR2 amplification of the full ORF2 and ORF3 regions. Nine extracts (9/19; 47.4%) yielded positive results and were subsequently subjected to NGS. An additional four samples (4/19; 21.1%) were successfully sequenced by conventional Sanger sequencing of amplicons spanning the partial ORF1/ORF2 region. From these, sequencing datasets were successfully generated, enabling the identification of four genotypes across two genogroups by the Norovirus Typing Tool: GI.6 (n = 2), GII.2 (n = 3), GII.4 (n = 6), and GII.7 (n = 2). On average, NGS sequencing produced 2.4 million reads, of which 3874 reads were specific to HuNoV, resulting in a mean coverage depth of 157×. Genotyping of the remaining six RT-qPCR-positive samples was unsuccessful, likely due to low viral loads (Cq > 36), which prevented successful amplification of both MLR2 and ORF1/ORF2. A detailed overview of HuNoV-positive RNA extracts successfully characterized by NGS and Sanger sequencing, including sample source, collection period, identified genotype, and corresponding GenBank accession numbers, is provided in Table 3.

**Figure 3:**
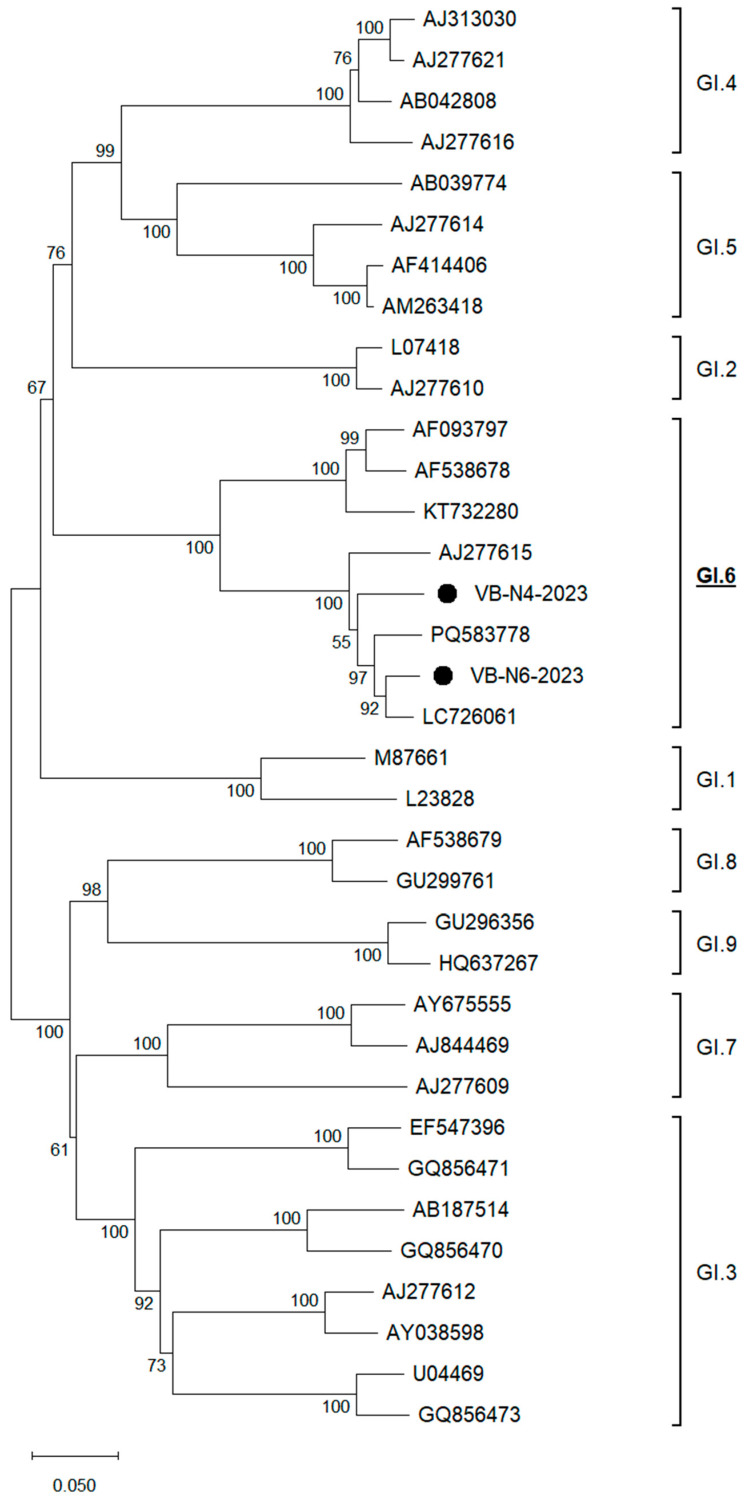
ORF2/ORF3-based unrooted phylogenetic tree of HuNoV GI strains identified in this study and reference strains recovered in the GenBank database. The Maximum Likelihood method and Tamura-Nei model (four parameters) with gamma distribution and invariable sites were used for the phylogeny. A total of 1000 bootstrap replicates were used to estimate the robustness of individual nodes on the phylogenetic tree. Black dots indicate strains detected in this study. The scale bar indicates nucleotide substitutions.

Phylogenetic analysis of GI genotype sequences revealed that Serbian isolate VB-N6-2023 clustered with isolate GI.6 LC726061 (node support 0.927), while VB-N4-2023 was positioned on a separate branch to the clade containing GI.6 PQ583778 and (VB-N6-2023, GI.6 LC726061) (Figure 3). The attachment of VB-N4-2023 to that clade had weak to moderate support (0.556), so its exact placement within that subcluster was somewhat uncertain, but was clearly inside GI.6. In terms of genotype diversity, the VB-N4-2023/VB-N6-2023 distance (≈0.076) was larger than the distance between VB-N6-2023 and GI.6 LC726061 (≈0.036), strengthening assumption that the VB-N6-2023 and VB-N4-2023 represent distinct GI.6 lineages. Sequence identity matrix analysis showed that, within genotype GI.6, the two Serbian isolates shared 93.4% nucleotide identity, and each was ≈91–92% identical to GI.6 AJ277615, but only ≈80% identical to GI.6 AF093797/AF538678, indicating significant intra-genotype diversity. This pattern is most consistent with ≥2 circulating GI.6 lineages in Serbian berries (i.e., multiple introductions), rather than a single lineage.

**Figure 4:**
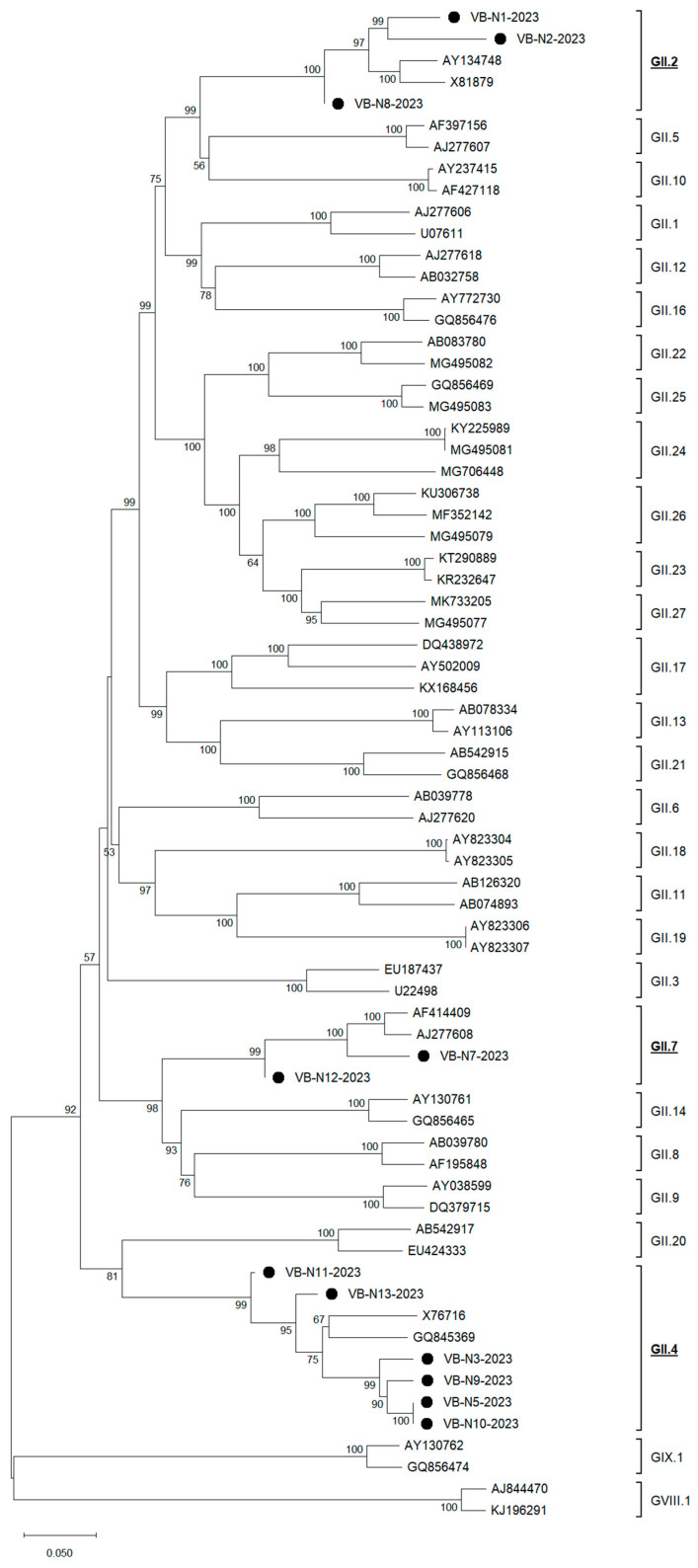
ORF2/ORF3-based unrooted phylogenetic tree of HuNoV GII strains identified in this study and reference strains recovered in the GenBank database. The Maximum Likelihood method and Tamura–Nei model (four parameters) with gamma distribution and invariable sites were used for the phylogeny. A total of 1000 bootstrap replicates were used to estimate the robustness of individual nodes on the phylogenetic tree. Black dots indicate strains detected in this study. The scale bar indicates nucleotide substitutions.

Within genotype GII, phylogenetic analysis identified that the two GII.2 reference strains AY134748 and X81879 formed a strongly supported clade (1.000) that was one of three primary lineages in the tree; the other two were VB-N1-2023 and a subclade containing VB-N2-2023 and VB-N8-2023 (support 0.631). VB-N2-2023 and VB-N8-2023 are closely related, but with markedly different terminal branch lengths (0.0647 vs. 0.0020 substitutions/site), indicating greater divergence in VB-N2-2023. Patristic distances among the Serbian GII.2 genotype isolates showed substantial heterogeneity: 0.0667 (VB-N2/VB-N8), 0.0497 (VB-N1/VB-N8), and 0.1124 (VB-N1/VB-N2), respectively. Sequence identity demonstrated that VB-N1-2023 showed 88.7–89.2% identity to the two aforementioned reference strains, consistent with its placement as a separate branch in the tree, and VB-N2-2023 was similarly related to the references (87.0–88.7% identity) and was closely related to VB-N8-2023 in the tree (node support 0.631). The topology, node support, and patristic distances indicate ≥2 co-circulating GII.2 lineages in the Serbian norovirus dataset, paralleling the pattern we observed for GI.6. (Figure 4).

The majority of HuNoV isolates from berries belonged to genotype GII.4. Phylogenetic analysis showed that the historical references X76716 and GQ845369 formed a pair (node support 0.676) that clustered with a Serbian HuNoV GII.4 subclade comprising VB-N3-2023, VB-N9-2023, and an identical sister pair VB-N5-2023/VB-N10-2023 (support 1.000 for the N5/N10 node; 0.903 for N9 with that pair; 0.998 for the full N3/(N9,(N5,N10)) cluster). The combined reference isolates and VB group was moderately supported (0.752). VB-N13-2023 was sister to that group (0.955), and VB-N11-2023 was sister to (VB-N13 + the rest) with strong support (0.998). Pairwise comparisons mirrored this phylogenetic structure. Within the N3/N5/N9/N10 cluster, identities were high (95.1–100%), including VB-N5/VB-N10 (100%), N5/N9 = 96.4%, N3/N5 = 95.6%, N3/N9 = 95.1%, N3/N10 = 95.6%, and N9/N10 = 96.4%. Against the references, this cluster showed 87.2–89.0% identity, consistent with a recent, closely related GII.4 sublineage. In contrast, VB-N11-2023 and VB-N13-2023 formed a second VB lineage (N11/N13 = 91.0% identity) that was substantially more divergent from outbreak-like quartets.

Finally, when it comes to HuNoV GII.7 isolates we have determined that the two reference strains (AF414409 and AJ277608) formed a tight pair, and VB-N7-2023 clustered with them with maximal support (1.000). VB-N12-2023 is sister to this three-taxon cluster with very strong support (0.999), and has a near-zero (slightly negative) terminal branch—an ML rounding artifact consistent with extremely low divergence over the analyzed region. Following patristic distances reflected this topology: VB-N7/AF414409 was ≈0.0852, VB-N7/AJ277608 was ≈0.0879, VB-N12/AF414409 was ≈0.0991, VB-N12/AJ277608 was ≈0.1018, and VB-N7/VB-N12 was ≈0.1005. The two reference GII.7 isolates differed by ≈0.0350. Thus, both VB isolates were clearly GII.7, but they were not each other’s closest relatives since VB-N7-2023 sits within the reference cluster, while VB-N12-2023 branches just outside it. This non-monophyly of N7 and N12 tips within GII.7, and together with the comparable N7,12-to-reference and N7-to-N12 distances is most consistent with ≥2 GII.7 introductions into the VB dataset rather than a single lineage.

The Serbian GI.6 obtained isolates from berries (VB-N4-2023 and VB-N6-2023) showed the highest nt identities of 99.7% and 98.1%, respectively, to isolates from Japan and the USA, with additional matches ranging from 97.3–97.9% (Table 4). Among the GII.2 isolates, nt identities ranged from 95.4% to 97.9% with isolates from the USA and Japan. The most prevalent GII.4 Serbian berries’ isolates exhibited 95.9–98.8% nt identity to isolates from Japan, Canada, Thailand, the Netherlands, and France. Finally, the GII.7 isolates (VB-N7-2023 and VB-N12-2023) showed 97.6–98.1% nt identity with Japanese and Dutch sequences. Detailed sequence identities, isolates, and geographic origins are summarized in Table 4.

## 4. Discussion

In this study, human norovirus was detected in 4.2% (CI 95%: 2.6–6.5%) of berry fruits marketed in Serbia, including HuNoV GI (0.9%) and HuNoV GII (3.3%), primarily in frozen products. Similar prevalence was obtained from a study by Di Cola et al. [48] indicating that 4.1% of berry samples in Argentina, primarily frozen strawberries, tested positive for norovirus or rotavirus. Significantly lower prevalence was determined in a study of the presence of foodborne viruses on fresh and frozen fruit in Canada, in which 0.83% of fresh raspberries and 0.37% of frozen raspberries were positive for HuNoV [6]. A more recent study also from Canada indicated that the prevalence observed for HuNoV GI was 1.28%, while for both HAV and HuNoV GII it was below 5% in RTE cranberries [49]. The determined HuNoV prevalence in berries from Serbia was higher than in UK, where Cook et al. determined that 2.3% samples of fresh raspberries were Norovirus-positive [12]. Most of the positively testing fresh raspberry samples were imported, but no predominance of a genogroup, or any seasonality, was observed. In the same study, frozen raspberries accounted for 3.6% Norovirus-positive samples. Torok et al. estimated risk associated with human norovirus in fresh Australian berries at retail and determined that prevalence of HuNoV in fresh strawberries and blueberries was estimated to be <2% [15]. In contrast, higher prevalence was established in China, where in a 2016–2017 survey of 900 frozen and 900 fresh berry samples for domestic consumption it was revealed that HuNoV RNA was detected in 9% of frozen berry samples and 12.1% of fresh berry samples [1]. In contrast, in this same study, no HuNoV RNA was detected in 677 samples of frozen berries for export. Miotti et al. estimated that the worldwide prevalence of HuNoV and HAV in berries was calculated at 2.12% (95% CI 1.74–2.59%), and no statistically differences were observed among the viral types or genogroup categories, although a significant influence of the sample condition (fresh or frozen) was observed in relation to the prevalence of HuNoV GII [4]. Sarvikivi et al. analyzed 14 samples of frozen raspberries implicated in an outbreak in Finland and detected Norovirus in 2 (14.3%) samples [50], and Mäde et al. analysed 11 samples of frozen raspberries implicated in an outbreak in Germany and found 7 (63.6%) to be positive [51]. Our earlier surveillance (2017–2020) of 2244 berry samples using ISO 15216-2 revealed even higher prevalence in Serbian berries (GI: 1.9%, GII: 6.1%, HAV: 0.18%) [31]. These findings confirm that frozen berries pose a higher risk than fresh berries, likely due to virus stability at low temperatures, extensive commingling, and complex supply chains [16].

Globally, genogroup II remains the predominant cause of norovirus infections [52,53], while genogroup I is more often associated with sporadic cases. Phylogenetic analysis revealed substantial genetic diversity of HuNoV in Serbian berries, consistent with multiple introductions. Within GI.6, isolates VB-N4-2023 and VB-N6-2023 represented distinct lineages, reflecting intra-genotypic diversity previously observed in Europe and Asia [54,55]. A similar pattern was seen in GII.2, where Serbian isolates formed at least two divergent lineages, consistent with global reports linking this genotype to recombinant variants and outbreaks [56,57]. Since 2012, GII.4 Sydney has been the predominant norovirus genotype worldwide [52,58], existing as two types: GII.4 Sydney[P31] and GII.4 Sydney[P16]. These two genotypes have consistently been the predominant strains in both outbreaks and sporadic cases of gastroenteritis globally. However, novel non-GII.4 genotypes have been increasingly documented, particularly GII.17[P17] in the winter season of 2014–2015 and GII.2[P16] in 2016–2017, causing increased HuNoV outbreaks in China and other countries [59]. Most isolates belonged to GII.4. One cluster (VB-N3, VB-N5, VB-N9, and VB-N10) showed high identity, suggesting local persistence, whereas VB-N11 and VB-N13 formed a distinct branch, indicating the presence of multiple GII.4 sublineages. For GII.7, isolates clustered with international references but appeared to reflect at least two independent introductions. The high nucleotide identities (95–99%) with reference strains from Asia, Europe, and North America suggest global circulation of lineages, likely facilitated by food trade and travel [60]. These findings indicate that berry contamination in Serbia does not arise from a single source but from multiple introductions involving different genotypes and sublineages. Both preharvest and postharvest contamination events appear plausible. This highlights the importance of molecular surveillance along the food supply chain and integration with clinical data to better trace transmission pathways.

Accurate prevalence estimates are challenged by methodological limitations, including PCR inhibition from berry-derived compounds (polyphenols and anthocyanins), low viral extraction efficiency, and losses of eluted virions during sample processing [31]. In this study, we employed a modified ISO 15216-2 standard method to enable efficient screening of several hundreds of samples for the presence or absence of HuNoV. While this method is still regarded as the gold standard [32], ongoing efforts by several researchers aim to optimize it further to improve performance [61,62,63]. As required by ISO standard, extraction efficiency was monitored using surrogate virus (Mengovirus) as a process control. However, Mengovirus recovery rates (1% to 10%) could not be applied for correcting norovirus concentrations which could potentially be 10–50 times higher, since these serve primarily as a quality assurance measure to verify extraction and amplification success. Moreover, control virus and the target virus differ in structure, surface properties, and likely contamination route, so their recovery efficiencies are not necessarily comparable. In addition, changes in extraction parameters could improve Mengovirus recovery while simultaneously reducing norovirus recovery. Thus, correcting norovirus concentrations on the basis of Mengovirus recovery would risk introducing greater uncertainty due to the surrogate’s limitations, rather than improving accuracy.

In this study, we used an RT-dPCR assay and successfully demonstrated the quantification of both HuNoV genogroups GI and GII RNA in almost all contaminated berry samples, previously screened as positive by gold-standard RT-qPCR. The assay was adapted from standardized RT-qPCR protocols [36,37,38,39,40], targeting the conserved ORF1-ORF2 junction with genogroup-specific primers and double-quenched probes to ensure broad coverage across genetically diverse strains. The principle of dividing molecules into distinct reactions like in RT-dPCR enhances sensitivity and inhibitor tolerance compared to conventional RT-qPCR, thereby minimizing competition between target and background [64]. Quantification in our case was consistent with low variation, although variability was concentration-dependent. Linearity was maintained down to approximately 10 genome copies/μL of in vitro-transcribed RNA, confirming assay sensitivity at low target levels. Global data on viral load in naturally contaminated berries are scarce, as most studies focus on method optimization using inoculated samples. Using RT-dPCR, HuNoV RNA in Serbian berries ranged from 34 to 105 gc/g for GI and 23 to 658 gc/g for GII, with median concentrations of 72 gc/g and 153 gc/g, respectively. These values were higher than previously reported for strawberries in Germany (average 8 ± 6 gc/g) [65], though the authors note that absolute genome copy numbers should be interpreted cautiously due to matrix effects and proximity to the method’s LOD. Comparatively, median viral loads in shellfish associated with illness were 123 gc/g for GI and 194 gc/g for GII [66]. Reported LODs for HuNoV in berries vary across studies, ranging from 20–40 gc/g [26] to 2.5 × 10^4^ copies/25 g using RT-ddPCR [67], highlighting matrix- and method-dependent variability. Boxmann et al. in an international interlaboratory study succeeded in achieving overall linearity of quantitative results down to an input of 100 gc/μL external amplification control RNA [68].

A key advantage of RT-dPCR over RT-qPCR is its capacity for absolute quantification without reliance on standard curves, reducing a major source of analytical variability [69,70]. RT-dPCR also shows increased tolerance to PCR inhibitors commonly co-extracted from complex food matrices, as demonstrated in previous studies with unfiltered berry RNA extracts [67,69,71]. These properties suggest that RT-dPCR can provide more reliable quantification in inhibitor-rich matrices. However, in our study we found two HuNoV GII samples that were positive in late Cq values (Cq > 38) while being negative in RT-dPCR, i.e., <LOD95 of 2.2 gc/g. We are of opinion that this pattern is expected near or below the detection limit since RT-qPCR can occasionally yield late positives below its respective LOD95, whereas RT-dPCR (despite a lower LOD95) may fail to register positives in a single well due to stochastic effects. Moreover, most digital PCR platforms do not analyze the full well. Typically, around 14–20 µL out of 40 µL worth of partitions are effectively read in the Qiacuity platform [72]. Applying Poisson statistics (P(0) = e^−λ^), the probability of obtaining an all-negative result at this effective input volume in our cases was between 1% and 11%, which is entirely plausible near LOD. Alternatively causes for these dubious cases could be attributed to subtle inhibition, invalid partitions, or adsorption/pipetting loss when dealing with ultra-low copy numbers. Similar remarks were reported by Larocque et al. [70] who adapted crystal digital RT-PCR (RT-cdPCR) for HuNoV GI/GII and HAV in frozen raspberries and reported that for HuNoV GI and HAV the RT-cdPCR assays had decreased qualitative sensitivity compared with the corresponding RT-qPCR assays (while HuNoV GII was more sensitive by cdPCR). Consistent with qPCR-positive/dPCR-negative discordance in part of our dataset, Coudray-Meunier et al. compared microfluidic RT-dPCR with RT-qPCR for HAV and HuNoV and found comparable or approximately 1-log-lower sensitivity for RT-dPCR for HuNoV GII RNA [73]. Also, Wilczek et al. found that, out of 128 plasma samples, there were 16 samples that were qRT-PCR-positive and RT-ddPCR-negative and some results did not demonstrate a clear superiority in sensitivity for RT-ddPCR [74].

The integration of RT-dPCR into routine food virology workflows, whether as a complement to RT-qPCR or as a standalone method, remains a subject of debate. On the one hand, RT-dPCR involves higher costs for equipment and reagents, as well as longer processing times [75]. Large-scale testing to improve HuNoV estimations in food is currently impractical due to the low throughput of dPCR, its operational complexity, and the fact that RT-dPCR reaction mixes are approximately twice as expensive as conventional RT-qPCR mixes [76,77]. Both RT-qPCR and RT-dPCR perform well within specific dynamic ranges (moderate to high viral loads) but are limited at extremely low nucleic acid concentrations, where RT-qPCR in particular struggles with sensitivity [78]. Sole reliance on RT-dPCR also carries risks, notably the potential overestimation of prevalence if stringent quality controls are not in place. This could lead to inflated positivity rates that may be misinterpreted as genuine increases in viral occurrence, with adverse consequences for industry compliance, marketability, and the costs of mitigation measures. In our view, other sources of variability beyond the choice of PCR platform are likely to contribute more substantially to uncertainty, and these should be systematically identified and addressed. A coordinated, peer-reviewed international study validating digital PCR for the detection of enteric viruses in berries similar to the work of Boxman et al. [68] on shellfish quantification would provide critical guidance for the field.

## 5. Conclusions

This study confirms the presence of HuNoV RNA in berry samples from Serbia, specifically in the Moravica and Zlatibor counties, which represent the country’s main raspberry and blackberry production regions. Four genotypes (GI.6, GII.2, GII.4, and GII.7) were detected, indicating the co-circulation of both genogroups across orchards, cold storage facilities, and local markets. The observed prevalence, especially high in frozen berries, and viral loads underscore a significant public health risk associated with the consumption of thermally untreated berry products. Continued surveillance of the molecular characteristics of circulating HuNoV strains is crucial for understanding source attribution and mitigating foodborne outbreaks, particularly in the context of international fruit trade.

## Figures and Tables

**Table 1:** Primers and probes used in the study.

Target	Name	Sequence	Reference
Norovirus GI	G1-fwd	CGC TGG ATG CGN TTC CAT	[36]
	G1-rev	CCT TAG ACG CCA TCA TCA TTT AC	[37]
	G1-probe	FAM-TGG ACA GGA GAY CGC RAT CT-BHQ1 ^1^	[37]
Norovirus GII	G2-fwd	ATG TTC AGR TGG ATG AGR TTC TCW GA	[38]
	G2-rev	TCG ACG CCA TCT TCA TTC ACA	[39]
	G2-probe	HEX-AGC ACG TGG GAG GGC GAT CG-BHQ1 ^1^	[38]
Mengovirus	Mengo-fwd	GCG GGT CCT GCC GAA AGT	[40]
	Mengo-rev	GAA GTA ACA TAT AGA CAG ACG CAC AC	[40]
	Mengo-probe	ROX-ATC ACA TTA CTG GCC GAA GC-MGBNFQ	[40]

^1^ ZEN™/Iowa Black^®^ FQ for RT-dPCR.

**Table 2:** HuNoV GI and GII viral load (mean, gc/g of berry), as determined by RT-qPCR and RT-dPCR.

Sample	HuNoV GI RT-qPCR (Cq)	HuNoV GI RT-dPCR (gc/g)	HuNoV GII RT-qPCR (Cq)	HuNoV GII RT-dPCR (gc/g)	Extraction Efficiency (%)
1	33.9	85	<LOD *	not tested	3.9
2	<LOD	not tested	34.4	63	3.5
3	<LOD	not tested	33.8	138	6.9
4	34.7	59	<LOD	not tested	7.4
5	<LOD	not tested	32.5	169	5.1
6	34.2	105	<LOD	not tested	1.3
7	<LOD	not tested	31.1	658	11.6
8	<LOD	not tested	37.1	25	8.9
9	<LOD	not tested	34.1	78	6.4
10	<LOD	not tested	31.7	328	4.4
11	36.2	34	<LOD	not tested	5.3
12	<LOD	not tested	36.5	23	2.6
13	<LOD	not tested	38.9	<RT-dPCR LOD95	4.6
14	<LOD	not tested	35.1	45	5.8
15	<LOD	not tested	33.4	193	7.2
16	<LOD	not tested	38.6	<RT-dPCR LOD95	1.6
17	<LOD	not tested	34.3	82	3.4
18	<LOD	not tested	32.8	218	3.7
19	<LOD	not tested	36.9	37	1.8

* RT-qPCR LOD95% for modified ISO 15216-2:2019 for HuNoV GI = 35 gc/g and HuNoV GII = 20 gc/g of berry.

**Table 3:** Summary of RNA samples positive for HuNoV GI and GII, as determined by capsid-targeted next-generation sequencing.

Isolate	Fruit Type	Fresh or Frozen	Period	Genotype	Accession No.
VB-N1-2023	Raspberries	Fresh	June 2023	GII.2	OR826789
VB-N2-2023	Raspberries	Fresh	July 2023	GII.2	PQ243047
VB-N3-2023	Blackberries	Frozen	August 2023	GII.4	PQ243256
VB-N4-2023	Raspberries	Frozen	July 2023	GI.6	OR816112
VB-N5-2023	Raspberries	Fresh	June 2023	GII.4	OR794161
VB-N6-2023	Raspberries	Frozen	August 2023	GI.6	PQ249007
VB-N7-2023	Raspberries	Frozen	July 2023	GII.7	OR821720
VB-N8-2023	Raspberries	Fresh	August 2023	GII.2	PQ844635
VB-N9-2023	Raspberries	Frozen	July 2023	GII.4	OR794162
VB-N10-2023	Blackberries	Frozen	July 2023	GII.4	OR794207
VB-N11-2023	Raspberries	Frozen	July 2023	GII.4	PQ844637
VB-N12-2023	Raspberries	Frozen	July 2023	GII.7	PQ844638
VB-N13-2023	Blackberries	Frozen	June 2023	GII.4	PQ844641

**Table 4:** Genetic matching of HuNoV sequences generated in this study. Sample ID, genotype, collection year and country, and the closest match from NCBI BLAST (accessed on 19 August 2025) are shown. Genotype classification follows NCBI and Norovirus Typing Tool assignments.

Sample ID	Genotype	Close Match Strain (Accession No.)	Country	% nt Identity
VB-N4-2023	GI.6	Hu/GI/JP/2008/GI.Pb-GI.6/HO-11 (LC122692)	Japan	99.7
VB-N6-2023	GI.6	G19_018 (MK789655)	France	97.9
VB-N1-2023	GII.2	Hu/US/2016/GII.P2-GII.2/Washington0526 (MK753007)	USA	97.9
VB-N2-2023	GII.2	Hu/US/2019/GII.2[P16]/CA-RGDS-1097 (MT738527.1)	USA	95.6
VB-N8-2023	GII.2	Hu/GII.2/HS255/2011/USA (KJ407074.2)	USA	97.6
VB-N3-2023	GII.4	Hu/GII/JP/2013/GII.Pe-GII.4/MI-17 (LC122792)	Japan	98.8
VB-N5-2023	GII.4	BMH19-097 (MW661264)	Canada	98.5
VB-N9-2023	GII.4	CU-PBH23217-STN (PP564826)	Thailand	98.5
VB-N10-2023	GII.4	RIVM-NOV-2018-0407_R03-13 (OP205558)	The Netherlands	98.7
VB-N11-2023	GII.4	G19_033 (MK907797)	France	97.6
VB-N13-2023	GII.4	Hu/GII.4/DBM15-156/2015/THA (MG786781)	Thailand	95.9
VB-N7-2023	GII.7	II.7[P7]/TKY2799/2024 (LC877023)	Japan	98.1
VB-N12-2023	GII.7	RIVM-NOV-2016-0195_R01-02 (OP205528)	The Netherlands	97.6

## Data Availability

Sequence data generated in this study have been shared via Genbank, the open access, annotated collection of all publicly available nucleotide sequences and their protein translations under the accession numbers listed in Table 3.

## Supplementary Materials

The following supporting information can be downloaded at https://www.mdpi.com/article/10.3390/foods14183257/s1, Table S1: Cq Values, Extraction Efficiency, and Inhibition in HuNoV Detection: ISO vs. Modified ISO; Table S2: Detection Rates and Viral Loads of HuNoV GI and GII in Berry Samples Assessed by RT-qPCR, RT-dPCR, and Capsid NGS.

## References

[1] Gao X., Wang Z., Wang Y., Liu Z., Guan X., Ma Y., Zhou H., Jiang Y., Cui W., Wang L. (2019). Surveillance of norovirus contamination in commercial fresh/frozen berries from Heilongjiang Province, China, using a TaqMan real-time RT-PCR assay. Food Microbiol..

[2] Cotterelle B., Drougard C., Rolland J., Becamel M., Boudon M., Pinede S., Traoré O., Balay K., Pothier P., Espié E. (2005). Outbreak of norovirus infection associated with the consumption of frozen raspberries, France, March 2005. Euro Surveill..

[3] Oteiza J.M., Prez V.E., Pereyra D., Jaureguiberry M.V., Sánchez G., Sant’Ana A.S., Barril P.A. (2022). Occurrence of Norovirus, Rotavirus, Hepatitis a Virus, and Enterovirus in Berries in Argentina. Food Environ. Virol..

[4] Miotti C., Signorini M.L., Oteiza J.M., Prez V.E., Barril P.A. (2024). Meta-analysis of the prevalence of norovirus and hepatitis a virus in berries. Int. J. Food Microbiol..

[5] Bozkurt H., Phan-Thien K.Y., van Ogtrop F., Bell T., McConchie R. (2021). Outbreaks, occurrence, and control of norovirus and hepatitis a virus contamination in berries: A review. Crit. Rev. Food Sci. Nutr..

[6] Steele M., Lambert D., Bissonnette R., Yamamoto E., Hardie K., Locas A. (2022). Norovirus GI and GII and hepatitis A virus in berries and pomegranate arils in Canada. Int. J. Food Microbiol..

[7] Raymond P., Paul S., Perron A., Bellehumeur C., Larocque É., Charest H. (2022). Detection and Sequencing of Multiple Human Norovirus Genotypes from Imported Frozen Raspberries Linked to Outbreaks in the Province of Quebec, Canada, in 2017. Food Environ. Virol..

[8] Elmahdy E.M., Shaheen M.N.F., Mahmoud L.H.I., Hammad I.A., Soliman E.R.S. (2022). Detection of Norovirus and Hepatitis A Virus in Strawberry and Green Leafy Vegetables by Using RT-qPCR in Egypt. Food Environ. Virol..

[9] Hall A.J., Wikswo M.E., Pringle K., Gould L.H., Parashar U.D. (2014). Vital signs: Foodborne norovirus outbreaks—United States, 2009–2012. MMWR Morb. Mortal. Wkly. Rep..

[10] Chatziprodromidou I.P., Bellou M., Vantarakis G., Vantarakis A. (2018). Viral outbreaks linked to fresh produce consumption: A systematic review. J. Appl. Microbiol..

[11] Huvarova V., Kralik P., Vasickova P., Kubankova M., Verbikova V., Slany M., Babak V., Moravkova M. (2018). Tracing of Selected Viral, Bacterial, and Parasitic Agents on Vegetables and Herbs Originating from Farms and Markets. J. Food Sci..

[12] Cook N., Williams L., D’Agostino M. (2019). Prevalence of Norovirus in produce sold at retail in the United Kingdom. Food Microbiol..

[13] De Keuckelaere A., Baert L., Stals A., De Vocht M., Li D., Delbeke S., Lauryssen S., Jacxsens L., Sas B., Uyttendaele M. (2018). Survey conducted by a consumer organization for the presence of bacterial and viral pathogens on high risk fresh produce from the Belgian market. Acta Hortic..

[14] Gao J., Xue L., Li Y., Zhang J., Dai J., Ye Q., Wu S., Gu Q., Zhang Y., Wei X. (2024). A systematic review and meta-analysis indicates a high risk of human noroviruses contamination in vegetable worldwide, with GI being the predominant genogroup. Int. J. Food Microbiol..

[15] Torok V.A., Hodgson K.R., Jolley J., Turnbull A., McLeod C. (2019). Estimating risk associated with human norovirus and hepatitis A virus in fresh Australian leafy greens and berries at retail. Int. J. Food Microbiol..

[16] Food and Agriculture Organization of the United Nations/World Health Organization Microbiological Hazards in Fresh Fruits and Vegetables. https://www.fao.org/fileadmin/templates/agns/pdf/jemra/FFV_2007_Final.pdf.

[17] Ekundayo T.C., Ijabadeniyi O.A. (2023). Human norovirus contamination challenge in fresh produce: A global prevalence and meta-analytic assessment. J. Appl. Microbiol..

[18] Šapić S., Jakšić M., Stojković D. (2020). The raspberry commodity exchange in Serbia: An exploratory research of producers’ attitudes. Ekon. Preduzeća.

[19] Statistical Yearbook of Serbia. https://publikacije.stat.gov.rs/G2024/Pdf/G20242057.pdf.

[20] European Commission RASFF Window. https://webgate.ec.europa.eu/rasff-window/screen/search.

[21] Atmar R.L., Opekun A.R., Gilger M.A., Estes M.K., Crawford S.E., Neill F.H., Graham D.Y. (2008). Norwalk Virus Shedding after Experimental Human Infection. Emerg. Infect. Dis..

[22] Atmar R.L., Opekun A.R., Gilger M.A., Estes M.K., Crawford S.E., Neill F.H., Ramani S., Hill H., Ferreira J., Graham D.Y. (2014). Determination of the 50% human infectious dose for Norwalk virus. J. Infect. Dis..

[23] Velebit B., Djordjevic V., Milojevic L., Babic M., Grkovic N., Jankovic V., Yushina Y. (2019). The common foodborne viruses: A review. IOP Conf. Ser. Earth Environ. Sci..

[24] Lee N., Chan M.C., Wong B., Choi K.W., Sin W., Lui G., Chan P.K., Lai R.W., Cockram C.S., Sung J.J. (2007). Fecal viral concentration and diarrhea in norovirus gastroenteritis. Emerg. Infect. Dis..

[25] Rzeżutka A., Cook N., Motarjemi Y. (2014). Viruses: Hepatitis A Virus. Encyclopedia of Food Safety.

[26] Raymond P., Paul S., Perron A., Deschênes L. (2021). Norovirus Extraction from Frozen Raspberries Using Magnetic Silica Beads. Food Environ. Virol..

[27] Summa M., Maunula L. (2018). Rapid Detection of Human Norovirus in Frozen Raspberries. Food Environ. Virol..

[28] Hedman J., Rådström P., Wilks M. (2013). Overcoming Inhibition in Real-Time Diagnostic PCR. PCR Detection of Microbial Pathogens.

[29] Dramé M., Tabue Teguo M., Proye E., Hequet F., Hentzien M., Kanagaratnam L., Godaert L. (2020). Should RT-PCR be considered a gold standard in the diagnosis of COVID-19?. J. Med. Virol..

[30] Oh C., Zhou A., O’Brien K., Schmidt Arthur R., Geltz J., Shisler Joanna L., Schmidt Arthur R., Keefer L., Brown William M., Nguyen Thanh H. (2023). Improved performance of nucleic acid-based assays for genetically diverse norovirus surveillance. Appl. Environ. Microbiol..

[31] Jaykus L.-A., Bidawid S., Bosch A., Butot S., Cook N., Gummalla S., Lowther J., Nasheri N., Pintó R.M., Schaffner D.W. (2026). Detection of foodborne viruses in berries—State of science and future considerations. Food Control.

[32] Mirmahdi R.S., Dicker S.L., Yusuf N.G., Montazeri N. (2025). Navigating Uncertainties in RT-qPCR and Infectivity Assessment of Norovirus. Food Environ. Virol..

[33] Kojabad A.A., Farzanehpour M., Galeh H.E.G., Dorostkar R., Jafarpour A., Bolandian M., Nodooshan M.M. (2021). Droplet digital PCR of viral DNA/RNA, current progress, challenges, and future perspectives. J. Med. Virol..

[34] Persson S., Eriksson R., Lowther J., Ellström P., Simonsson M. (2018). Comparison between RT droplet digital PCR and RT real-time PCR for quantification of noroviruses in oysters. Int. J. Food Microbiol..

[35] (2019). Microbiology of the Food Chain: Horizontal Method for Determination of Hepatitis A Virus and Norovirus Using Real-Time RT-PCR. Part 2: Method for Detection.

[36] da Silva A.K., Le Saux J.C., Parnaudeau S., Pommepuy M., Elimelech M., Le Guyader F.S. (2007). Evaluation of removal of noroviruses during wastewater treatment, using real-time reverse transcription-PCR: Different behaviors of genogroups I and II. Appl. Environ. Microbiol..

[37] Svraka S., Duizer E., Vennema H., de Bruin E., van der Veer B., Dorresteijn B., Koopmans M. (2007). Etiological role of viruses in outbreaks of acute gastroenteritis in The Netherlands from 1994 through 2005. J. Clin. Microbiol..

[38] Loisy F., Atmar R.L., Guillon P., Le Cann P., Pommepuy M., Le Guyader F.S. (2005). Real-time RT-PCR for norovirus screening in shellfish. J. Virol. Methods.

[39] Kageyama T., Kojima S., Shinohara M., Uchida K., Fukushi S., Hoshino F.B., Takeda N., Katayama K. (2003). Broadly reactive and highly sensitive assay for Norwalk-like viruses based on real-time quantitative reverse transcription-PCR. J. Clin. Microbiol..

[40] Pintó R.M., Costafreda M.I., Bosch A. (2009). Risk assessment in shellfish-borne outbreaks of hepatitis A. Appl. Environ. Microbiol..

[41] Wilrich C., Wilrich P.-T. (2019). Estimation of the POD Function and the LOD of a Qualitative Microbiological Measurement Method. J. AOAC Int..

[42] Forootan A., Sjöback R., Björkman J., Sjögreen B., Linz L., Kubista M. (2017). Methods to determine limit of detection and limit of quantification in quantitative real-time PCR (qPCR). Biomol. Detect. Quantif..

[43] Parra G.I., Squires R.B., Karangwa C.K., Johnson J.A., Lepore C.J., Sosnovtsev S.V., Green K.Y. (2017). Static and Evolving Norovirus Genotypes: Implications for Epidemiology and Immunity. PLoS Pathog..

[44] Kroneman A., Vennema H., Deforche K., v d Avoort H., Peñaranda S., Oberste M.S., Vinjé J., Koopmans M. (2011). An automated genotyping tool for enteroviruses and noroviruses. J. Clin. Virol..

[45] Kojima S., Kageyama T., Fukushi S., Hoshino F.B., Shinohara M., Uchida K., Natori K., Takeda N., Katayama K. (2002). Genogroup-Specific PCR Primers for Detection of Norwalk-Like Viruses. J. Virol. Methods.

[46] Kumar S., Stecher G., Suleski M., Sanderford M., Sharma S., Tamura K. (2024). Molecular Evolutionary Genetics Analysis Version 12 for Adaptive and Green Computing. Mol. Biol. Evol..

[47] Pierson-Perry J.F., Vaks J.E., Vore T., Durham A., Fischer C., Gutenbrunner C., Hillyard D., Kondratovich M., Ladwig P., Middleberg R. (2012). EP17-A2: Evaluation of Detection Capability for Clinical Laboratory Measurement Procedures; Approved Guideline.

[48] Di Cola G., Prez V.E., Fantilli A.C., Frydman C., Mozgovoj M., Luque L., Ferreyra L., Nates S.V., Pisano M.B., Ré V.E. (2025). Detection and genotyping of enteric foodborne viruses with high (NoV, HAV, HEV) and low (RV, AdV) health impact in ready-to-eat leafy vegetables and berries from Córdoba, Argentina. medRxiv.

[49] Chatonnat E., Manseau-Ferland K., Jubinville E., Goulet-Beaulieu V., Jean J. (2023). Prevalence of Foodborne Viruses in Berries Harvested in Canada. Foods.

[50] Sarvikivi E., Roivainen M., Maunula L., Niskanen T., Korhonen T., Lappalainen M., Kuusi M. (2012). Multiple norovirus outbreaks linked to imported frozen raspberries. Epidemiol. Infect..

[51] Mäde D., Trübner K., Neubert E., Höhne M., Johne R. (2013). Detection and Typing of Norovirus from Frozen Strawberries Involved in a Large-Scale Gastroenteritis Outbreak in Germany. Food. Environ. Virol..

[52] Carlson K.B., Dilley A., O’Grady T., Johnson J.A., Lopman B., Viscidi E. (2024). A narrative review of norovirus epidemiology, biology, and challenges to vaccine development. Vaccines.

[53] Desai R., Hembree C.D., Handel A., Matthews J.E., Dickey B.W., McDonald S., Hall A.J., Parashar U.D., Leon J.S., Lopman B. (2012). Severe Outcomes Are Associated with Genogroup II Genotype 4 Norovirus Outbreaks: A Systematic Literature Review. Clin. Infect. Dis..

[54] Leshem E., Barclay L., Wikswo M., Vega E., Gregoricus N., Parashar U.D., Vinjé J., Hall A.J. (2013). Genotype GI.6 Norovirus, United States, 2010–2012. Emerg. Infect. Dis..

[55] Singh A.K., Nagar J., Tandekar A., Singh S., Diwan V., Ravindran G.C., Tiwari R.R., Mishra P.K., Nema R.K. (2025). The evolving landscape of Norovirus GII genotypes in Asia: A systematic review and meta-analysis. J. Clin. Virol..

[56] Thongprachum A., Okitsu S., Khamrin P., Maneekarn N., Hayakawa S., Ushijima H. (2017). Emergence of Norovirus GII.2 and Its Novel Recombination during the Gastroenteritis Outbreak in Japanese Children in Mid-2016. Infect. Genet. Evol..

[57] Buesa J., Rodríguez-Díaz J., Galiana C., Villar L.M., Pérez-Rodríguez F.J., García-Durán F., Sánchez G. (2021). Epidemiology of GII.4 and GII.2 Norovirus Outbreaks in Closed and Semi-Closed Institutions in 2017 and 2018. J. Med. Virol..

[58] Jin M., Zhou Y.K., Xie H.P., Fu J.G., He Y.Q., Zhang S., Jing H.B., Kong X.Y., Sun X.M., Li H.Y. (2016). Characterization of the New GII.17 Norovirus Variant That Emerged Recently as the Predominant Strain in China. J. Gen. Virol..

[59] Bidalot M., Théry L., Kaplon J., De Rougemont A., Ambert-Balay K. (2017). Emergence of New Recombinant Noroviruses GII.P16-GII.4 and GII.P16-GII.2, France, Winter 2016 to 2017. Euro Surveill..

[60] Kambhampati A.K., Calderwood L., Wikswo M.E., Barclay L., Mattison C.P., Balachandran N., Vinjé J., Hall A.J., Mirza S.A. (2023). Spatiotemporal Trends in Norovirus Outbreaks in the United States, 2009–2019. Clin. Infect. Dis..

[61] Raymond P., Blain R., Nasheri N. (2025). Detection of Foodborne Viruses in Dates Using ISO 15216 Methodology. Viruses.

[62] Wang D., Cao J., Tian Z., Fang B., Qi X., Lei Z., Liu L., Zhu J., Ma L. (2022). Development of a New Concentration Method for Hepatitis A Virus Detection (ISO 15216–2:2019) in Manila Clams (*Ruditapes philippinarum*). LWT.

[63] Boudaud N., Chevaliez S., Cappelier J.-M., Le Guyader F.S., Gantzer C. (2021). Assessment of ISO Method 15216 to Quantify Hepatitis E Virus in Bottled Water. Food Environ. Virol..

[64] Quan P.-L., Sauzade M., Brouzes E. (2018). dPCR: A Technology Review. Sensors.

[65] Bartsch C., Höper D., Mäde D., Johne R. (2018). Analysis of frozen strawberries involved in a large norovirus gastroenteritis outbreak using next generation sequencing and digital PCR. Food Microbiol..

[66] Rexin D., Kaas L., Langlet J., Croucher D., Hewitt J. (2024). Droplet Digital PCR for Precise Quantification of Human Norovirus in Shellfish Associated with Gastroenteritis Illness. J. Food Prot..

[67] Sun C., Chen J., Li H., Fang L., Wu S., Jayavanth P., Tang S., Sanchez G., Wu X. (2021). One-step duplex RT-droplet digital PCR assay for the detection of norovirus GI and GII in lettuce and strawberry. Food Microbiol..

[68] Boxman I.L.A., Molin R., Persson S., Juréus A., Jansen C.C.C., Sosef N.P., Le Guyader S.F., Ollivier J., Summa M., Hautaniemi M. (2024). An international inter-laboratory study to compare digital PCR with ISO standardized qPCR assays for the detection of norovirus GI and GII in oyster tissue. Food Microbiol..

[69] Fraisse A., Coudray-Meunier C., Martin-Latil S., Hennechart-Collette C., Delannoy S., Fach P., Perelle S. (2017). Digital RT-PCR method for hepatitis A virus and norovirus quantification in soft berries. Int. J. Food Microbiol..

[70] Larocque É., Lévesque V., Lambert D. (2022). Crystal digital RT-PCR for the detection and quantification of norovirus and hepatitis A virus RNA in frozen raspberries. Int. J. Food Microbiol..

[71] Rački N., Morisset D., Gutierrez-Aguirre I., Ravnikar M. (2014). One-step RT-droplet digital PCR: A breakthrough in the quantification of waterborne RNA viruses. Anal. Bioanal. Chem..

[72] Bussmann M., Hesse M., Karalay O., Nash R., Missel A. (2022). Impact of Template Addition Volume and Analyzed Volume on Digital PCR Sensitivity.

[73] Coudray-Meunier C., Fraisse A., Martin-Latil S., Guillier L., Delannoy S., Fach P., Perelle S. (2015). A Comparative Study of Digital RT-PCR and RT-qPCR for Quantification of Hepatitis A Virus and Norovirus in Lettuce and Water Samples. Int. J. Food Microbiol..

[74] Wilczek M., Mańkowska-Wierzbicka D., Różańska A., Kalinowska A., Buda A., Pieszak M., Drzewiecka H., Mozer-Lisewska I. (2022). Droplet Digital PCR or Real-Time PCR as a Method for Quantifying SARS-CoV-2 RNA in Plasma—Is There a Difference?. Viruses.

[75] Youssfi W., Zhang W. (2025). Comparison of RT-qPCR and RT-ddPCR on Assessing Model Viruses in Wastewater. Water Environ. Res..

[76] Verhaegen B., De Reu K., De Zutter L., Verstraete K., Heyndrickx M., Vlaemynck G. (2016). Comparison of Droplet Digital PCR and qPCR for the Quantification of Shiga Toxin-Producing *Escherichia coli* in Bovine Feces. Toxins.

[77] Zhang H., Cao L., Brodsky J., Gablech I., Xu F., Li Z., Korabecna M., Neuzil P. (2024). Quantitative or Digital PCR? A Comparative Analysis for Choosing the Optimal One for Biosensing Applications. TrAC Trends Anal. Chem..

[78] Hedman J., Lavander M., Salomonsson E.N., Löfström C., Rådström P. (2018). Validation Guidelines for PCR Workflows in Bioterrorism Preparedness, Food Safety and Forensics. Accred. Qual. Assur..

