# Supplementary material for: Detection of Norovirus from Berries in Serbia by Digital PCR and NGS
Source: Foods. 2025 Sep 19;14(18):3257. doi: 10.3390/foods14183257 (PMC12469564; doi:10.3390/foods14183257)
Supplement: Supplementary file 1 [file foods-14-03257-s001.zip › foods-3878746-supplementary/Supplementary Table S1.pdf]

Supplementary Table S1. Cq Values, Extraction Efficiency, and Inhibition in HuNoV Detection: ISO vs. Modified ISO.

The mISO method improves upon ISO 15216-2:2019 by increasing the volume of elution buffer, incorporating polyvinylpyrrolidone (to mitigate the inhibitory effects of polyphenols present in berries), and employing a fluorocarbon fluid to selectively separate norovirus from host cells and other biological debris, followed by resin column-based viral RNA purification and concentration. Certified reference material in the form of lenticules produced by the United Kingdom Health Security Agency was used for this purpose.

HuNoV (gc/g)	Cq value		Extraction efficiency (%)		Inhibition (%)	
	ISO	mISO	ISO	mISO	ISO	mISO
$10^{2.7}$	32.86 ± 0.64	31.18 ± 0.38	2.86 ± 0.55	6.62 ± 0.83	< 75 (diluted)	< 75
$10^{2.2}$	35.07 ± 1.14	32.84 ± 1.28	3.45 ± 0.75	7.61 ± 2.88	< 75 (diluted)	< 75
$10^{1.7}$	36.97 ± 2.38	34.50 ± 1.77	3.73 ± 0.89	7.58 ± 3.74	< 75	< 75
$10^{1.2}$	nd	36.16 ± 2.13	1.56 ± 0.65	4.32 ± 1.31	< 75	< 75
$10^{0.6}$	nd	nd	nd	nd	nd	nd

*nd - not detected
