# Supplementary material for: Detection of Norovirus from Berries in Serbia by Digital PCR and NGS
Source: Foods. 2025 Sep 19;14(18):3257. doi: 10.3390/foods14183257 (PMC12469564; doi:10.3390/foods14183257)
Supplement: Supplementary file 1 [file foods-14-03257-s001.zip › foods-3878746-supplementary/Supplementary Table S2.pdf]

Supplementary Table S2. Detection Rates and Viral Loads of HuNoV GI and GII in Berry Samples Assessed by RT-qPCR, RT-dPCR, and NGS.

Samples	N	RT-qPCR			RT-dPCR (gc/g)		NGS	
		GI positive	GII positive	**TP**	**GI median**	**GII median**	**GI**	**GII**
Fresh Raspberries	272	1	6	7 (2.6%)	34	154	nd	4
Fresh Blackberries	70	nd	nd	nd	nt	nt	nt	nt
Frozen Raspberries	86	3	6	9 (10.5%)	85	136	2	4
Frozen Blackberries	22	nd	3	3 (13.6%)	nd	37	nd	3
TOTAL	450	4 (0.9%)	15 (3.3%)	19 (4.2%)	4	13	2	11

* TP – total positive per fruit/status,
nd – not detected,
nt – not tested
